# Stability and Pseudocatecholase Activity of Artificial Bis-Histidyl Copper Peptides

**DOI:** 10.1021/acs.inorgchem.5c02080

**Published:** 2025-10-08

**Authors:** Chiara Bottoni, Matteo Tegoni, Valentina Borghesani

**Affiliations:** Department of Chemistry, Life Sciences, and Environmental Sustainability, 9370 University of Parma, 43123 Parma, Italy

## Abstract

In this paper, 4-methylcatechol (4-MC) and L/D-Dopa are selected as target substrates for *de novo* protein design, serving as a proof-of-concept system for evaluating the catalytic activity of metal coordination sites. We report two novel water-soluble bis-histidyl peptides that exhibit catalytic activity, where structural constraints around the active site would play a pivotal role in metalloenzyme development. The particular tandem His–His motif in the peptides and favorable *E*_0_′ enable them to catalyze catechol oxidation reactions efficiently. The coordination behavior of the peptides with Cu­(II) and Cu­(I) ions is thoroughly investigated using a combination of analytical techniques, including potentiometric titration and fluorescence, ultraviolet–visible (UV–vis), and circular dichroism (CD) spectroscopies. The insights gained into the catalytic binding site and associated pseudocatecholase activity of these peptides contribute to the development of copper-based bioinspired artificial metalloenzymes.

## Graphical abstract

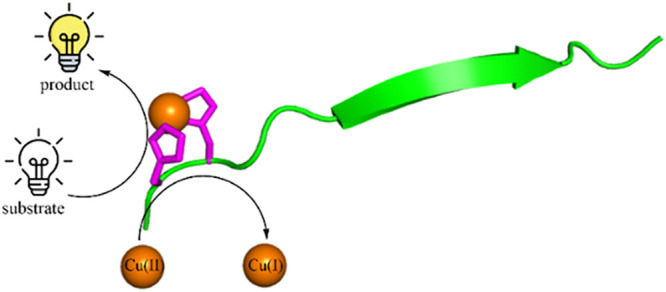

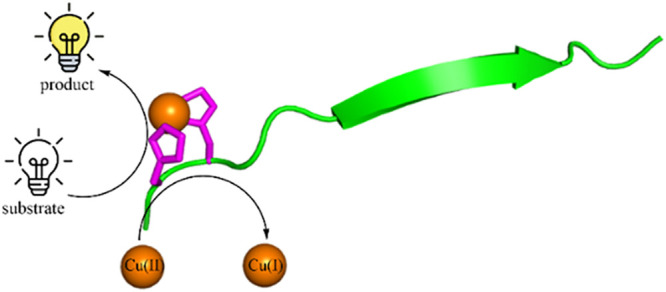

## Introduction

Metalloproteins promote several of the most complex biomolecular processes in nature. Through protein design, a large number of tailored peptides and proteins that either do not exist or have different functions compared to those present in nature have been developed in the last few decades.^1–8^

The chemistry of metallopeptides with different coordinating residues involved in metal coordination has also been extensively explored in the past years. The case of histidine residues is paradigmatic, as peptides containing this residue exhibit strong affinities for numerous transition metal ions. One interesting feature is that the affinity and redox properties of metal/peptide adducts vary with the number and position of histidine residues in the peptide sequence and the nature of the metal. Monohistidine peptides are so far the best characterized in terms of their interaction with metal ions, which are remarkable stable when histidine is located close to or at the peptide N-terminus.^9–11^ When multiple His residues are present in the sequence, the study of metal binding becomes more challenging, as small differences in the peptide sequences may result in large changes in the structure, stability, and function of metal complexes. However, it was widely demonstrated that in these systems, histidine residues act as anchor sites for metal ions.^9,12–15^

In recent years, it has become increasingly evident that His–His motifs are not only important for Cu­(II) binding but also, perhaps unexpectedly, exhibit an extraordinary affinity for Cu­(I). This evidence turns out to be highly relevant in the mechanism study of Cu­(II/I) binding to peptides associated with neurodegeneration (es. prion proteins, Aβ, and Tau peptides).^16–18^ However, although His–His peptides have been extensively studied, the stability of Cu­(I) adducts in conjunction with their catalytic aspects has been quite scarcely studied.

Recently, we reported a new artificial metalloprotein based on the SpyTag/SpyCatcher construct that has been provided with a Cu­(II)/ATCUN metal site designed on the SpyTag peptidic fragment.^19^ The ATCUN site (amino terminal Cu and Ni binding site) indeed is characterized by an H_2_N-Xxx-Xxx-His sequence at the N-terminus, which presents a high affinity for divalent copper and nickel. This artificial metal site, when mounted on the reconstituted final protein, has the property of promoting the formation of reactive oxygen species (ROS) in the presence of ascorbate.

In view of designing new catalytic artificial proteins using the construct Spy, we investigated the stability and pseudocatecholase activity of opper complexes formed with two minimalistic versions of SpyTag peptides containing a His–His dyad (Pep1 and Pep2, Figure 1). Both peptides are acetylated at the N-terminus, and therefore, the (His)_2_ site is expected to act as the primary binding site for both Cu­(I) and Cu­(II). A third peptide analog of Pep1, but devoid of His residues, has also been examined as a control peptide (PepCtrl). Finally, QIVMVDAYKG corresponds to the SpyTag sequence that allows for the lock-and-key interaction with the SpyCatcher counterpart. The last part of the sequence, after the consensus one, is AYKG. This sequence differs from AYKRYK, which is the canonical SpyTag C-term sequence. The absence of one tyrosine, one lysine, and one arginine is expected to simplify the study of copper binding to the His–His site. In particular, the absence of the KRYK sequence at the C-terminus much simplifies the potentiometric profile and, more broadly, the coordination study. However, its absence, if on the one hand makes it possible to clearly study the coordination environment around physiological pH, on the other hand, it does not allow the peptide to recombine with the protein.

**Figure 1:**
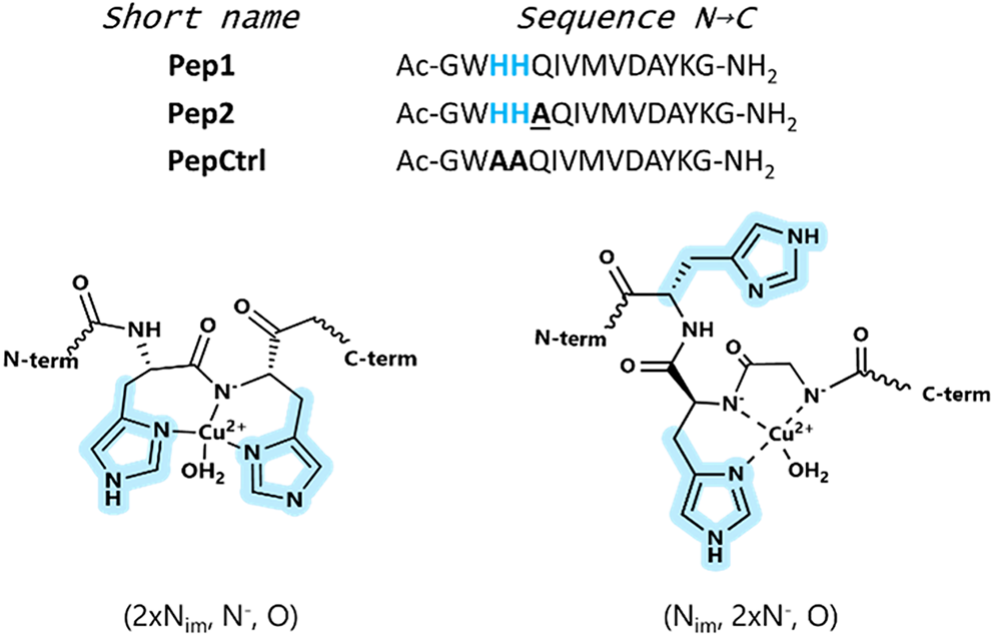
Sequences of peptides used in this study (sequence N → C) and schematic representation of the relevant copper­(II) coordination in [Cu­(LH)]^+^ (left) and [CuL] (right) species of Pep1 or Pep2 (L) at pH 7.4.

The interaction of these peptides with their protein counterparts is beyond the scope of this work. Rather, here, we wish to shed light on the binding capabilities of the His–His dyad upstream of the SpyTag system toward Cu­(I) and Cu­(II), the spectroscopic features of the copper­(II) complexes, and the capacity of these peptides to oxidize catechols. Also, the two peptides differ by one alanine residue placed between the (His)_2_ site and SpyTag sequence. The presence of this alanine residue in Pep2 was predicted to reduce steric hindrance and possibly modulate the presence of second–shell interactions around the copper-binding site. The speciation of the copper­(II) complexes with both peptides studied by potentiometry is presented here, along with the spectroscopic study of copper­(II) and copper­(I) adducts. Finally, the oxidation of 4-methylcatechol (4-MC) and L- and D-Dopa in the presence of copper and peptides was investigated. Due to the intrinsic chiral nature of the peptides, it is expected that the activated complex with the two enantiomers of Dopa could bring enantiomeric discrimination. Although we did not observe enantiomeric discrimination using the two isomers of Dopa, we still observed a slight difference in the stability of the copper adducts with the two different peptides.

## Results

### Peptide Protonation Equilibria

The protonation constants of the three peptides (Figure 1) were determined in aqueous solution by potentiometric titrations. The data are presented in Table 1, and the corresponding species distribution diagrams are shown in the Supporting Information (Figure S1). Pep1 and Pep2, in their fully protonated forms, are pentaprotic acids ([H_5_L]^3+^). The p*K*_a_ values determined by potentiometric titration are in agreement with the primary sequences of the peptides (Table 1). These dissociation processes correspond, in the first approximation, to the deprotonation of the carboxylic group of Asp, the imidazolium groups of two His, the hydroxyl group of Tyr, and the amino group of Lys. The stepwise deprotonation processes are shown in Scheme S1. It is worth noting in this context that the proton dissociation macroconstants reported in Table 1 are close in their value, indicating the presence of multiple and simultaneous proton dissociation microequilibria. As a consequence, the correspondence between a macroconstant and the proton dissociation of one specific functional group is an approximate description of these processes. This approach is, however, very common in the thermodynamic characterization of peptides: under this approximation, each macroconstant is associated with the functional group that most significantly contributes to that stepwise proton dissociation equilibrium.^12,20,21^

**Table 1:** Stability Constants (log β) and Proton Dissociation Constants (p*K*_a_) of the Fully Protonated Pep1 and Pep2 Peptides ([H5L]^3+^) and PepCtrl Peptide ([H3L]^+^) in Aqueous Solution (*T* = 298.2 K, *I* = 0.1 M of KCl)^a^

	Pep1 [H_5_L]^3+^			Pep2 [H_5_L]^3+^			PepCtrl [H_3_L]^+^		
species	log β	p*K*_a_		log β	p*K*_a_		log β	p*K*_a_	
[HL]^−^	10.10(9)	10.10(9)	NH_3_^+^ Lys	10.2(1)	10.16(5)	NH_3_^+^ Lys	9.88(6)	9.88(6)	NH_3_^+^ Lys
[H_2_L]	19.62(7)	9.51(7)	OH^–^ Tyr	19.9(1)	9.7(1)	OH^–^ Tyr	18.94(3)	9.06(3)	OH^–^ Tyr
[H_3_L]^+^	26.38(7)	6.77(7)	N_im_H^+^ His	26.50(9)	6.6(1)	N_im_H^+^ His	22.30(4)	3.90(4)	COOH Asp
[H_4_L]^2+^	32.18(8)	5.77(8)	N_im_H^+^ His	32.0(1)	5.5(1)	N_im_H^+^ His	– – –	– – –	– – –
[H_5_L]^3+^	35.83(4)	3.65(4)	COOH Asp	35.67(5)	3.67(5)	COOH Asp	– – –	– – –	– – –

a Standard deviations are given in parentheses. The acidic groups principally involved in each proton dissociation are also reported.

PepCtrl ([H_3_L]^+^) is devoid of His residues and consistently presents only 3 protonation constants. For this peptide, the p*K*_a_ values correspond to the deprotonation equilibria of the carboxylic group of Asp, the hydroxyl group of Tyr, and the amino group of Lys (Table 1). For all three peptides, the predominant form at pH 7.4 is [H_2_L], which implies that for both Pep1 and Pep2, at pH 7.4, both His residues are deprotonated (neutral imidazole form).

### Copper­(II)/Peptide Complex Formation Equilibria

The formation of copper­(II) complexes of Pep1 and Pep2 was studied by potentiometric titrations in the presence of a slight excess of ligand (Cu:L 1:1.2). For these stoichiometric ratios, only the formation of mononuclear complexes was observed. Titrations of systems containing Cu:Pep in a 1.6:1 ratio were attempted with the aim of excluding the formation of bimetallic complexes. However, precipitation was observed, which is consistent with that observed by ultraviolet–visible (UV–vis) Cu­(II) titration of the peptide at pH 7.4 (see below). The speciation models of the two systems are presented in Table 2. A representative distribution diagram for Pep1 and Pep2 is shown in Figure 2. Both peptides form seven monometallic complex species that differ in their protonation states.

**Figure 2:**
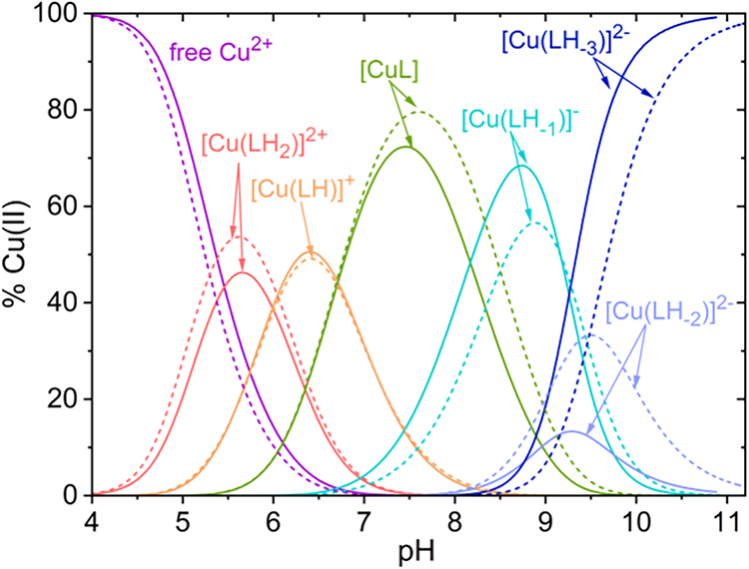
Representative distribution diagrams of the Cu­(II)/Pep1 (solid lines) and Pep2 (dotted lines) systems. *C*_Cu(II)_ = 0.25 mM, Cu:L = 1:1.2, *T* = 298.2 K, and *I* = 0.1 M (KCl).

**Table 2:** Logarithms of the Overall Stability Constants (log β) of the Copper­(II) Complexes of PepX (*X* = 1 or 2) in Aqueous Solution (*T* = 298.2 K, *I* = 0.1 M in KCl)^a^

species	Pep1 [H_5_L]^3+^	Pep2 [H_5_L]^3+^
[Cu(LH_2_)]^2+^	25.75(10)	25.6(2)
[Cu(LH)]^+^	19.73(15)	19.7(2)
[Cu(L)]	13.07(14)	12.9(3)
[Cu(LH_–1_)]^−^	4.9(2)	4.4(4)
[Cu(LH_–2_)]^2–^	–4.8(4)	–5.0(4)
[Cu(LH_–3_)]^2–^	–13.68(19)	–14.4(3)

a Standard deviations are given in parentheses.

The predominant species at around neutral pH is [CuL] (pH range 6.7–8.2), which accounts for ∼70% of the total copper at pH 7.4 (Figure 2). Below this pH range, the most abundant species are [Cu­(LH_2_)]^2+^ and [Cu­(LH)]^+^ with formation maxima at pH 5.6 and 6.4, respectively. Above pH 7.8, [Cu­(LH_–1_)]^−^ becomes the most abundant species, reaching its maximum at pH 8.8 (ca. 60% total copper). The last two deprotonation steps led to the formation of [Cu­(LH_–2_)]^2–^ and [Cu­(LH_–3_)]^3–^. At pH 7.4, at which the oxidation of catechols was studied, the speciation of the Pep1:Cu­(II) complex was 72% [CuL], 15% [Cu­(LH)]^+^, and 12%[Cu­(LH_–1_)]^−^, while that with Pep2 was 79% [CuL], 15% [Cu­(LH)]^+^, and 6%[Cu­(LH_–1_)]^−^.

Visible absorption and CD spectra of the Cu­(II)/PepX systems (*X* = 1 or 2) were collected at different pH values. The visible spectra of the Cu­(II)/Pep1 and Cu­(II)/Pep2 systems are shown in Figure 3. The CD spectra of both systems are provided in Supporting Information (Figure S2). These experimental data were treated with the HypSpec2014 software using the complex formation constants obtained by potentiometry as fixed parameters, allowing the molar absorption spectra of the single complex species to be obtained (Figure S6). The wavelengths of maximum absorption for each species are reported in Table S2 for ease of comparison between the experimental and calculated ones. The molar absorption spectra of some species, such as [Cu­(LH_–2_)]^2–^, were poorly determined since the amount of these species was low at the pH at which the spectra were collected.^22,23^

**Figure 3:**
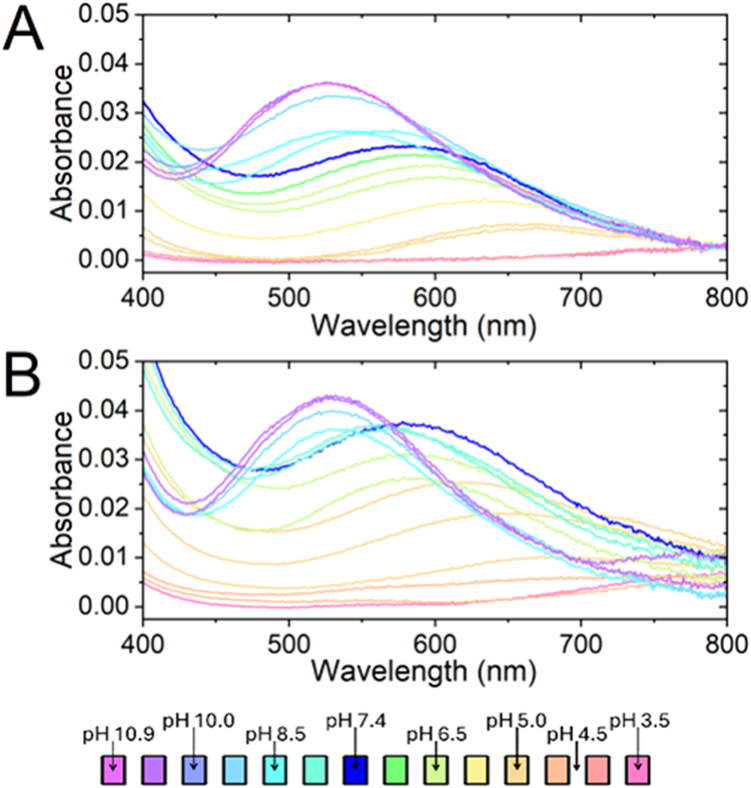
UV–vis spectra for the pH titration of Cu­(II):PepX systems (Pep1 in panel (A) and Pep2 in panel (B)). Cu/PepX = 1:1.25 at *T* = 25 °C and *I* = 0.1 M (KCl). C_Cu_ = 0.32 mM and [PepX] = 0.4 mM.

On the basis of the spectroscopic data and taking into account the Billo’s parameters,^22,23^ the coordination environment of copper­(II) as a function of pH can be proposed, as reported in Table 2. The spectra at pH 7.4 present a band with a λ_max_ of *ca*. 584 nm associated with the predominant formation of [Cu­(L)]. Considering Billo’s parameters,^22,23^ the groups coordinated on the equatorial plane of Cu­(II) are likely one imidazole nitrogen, two deprotonated peptide nitrogen atoms, and a water molecule (expected λ_max_ = 588 nm). Above pH 9, the absorption maximum shifts to 523 nm and remains constant until pH 11. In the pH range 9–11, the three species [Cu­(LH_–1_)]^−^, [Cu­(LH_–2_)]^2–^, and [Cu­(LH_–3_)]^3–^ are formed. The lack of shift in the absorption maximum suggests that the three species do not differ in the coordination environment of Cu­(II). The three species, therefore, differ only in the deprotonation states of the Tyr OH and the Lys amino group. Indeed, consistent with Billo’s parameters for all three species, Cu­(II) is coordinated on the equatorial plane by one imidazole and three peptide nitrogen atoms, as shown in Figure 4 for [Cu­(LH_–1_)]^−^ (expected λ_max_ = 523 nm).

**Figure 4:**
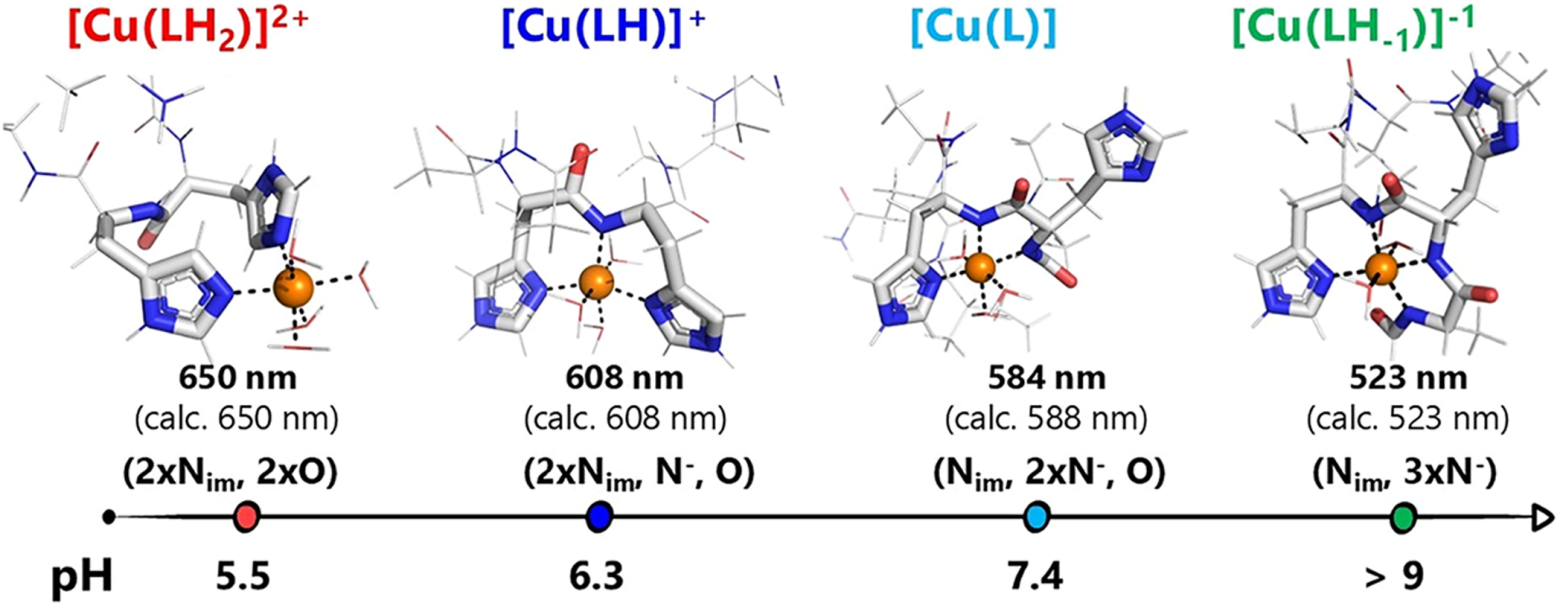
Representation of copper­(II) coordination modes as a function of pH.

At pH values below neutrality, the two major species are [Cu­(LH_2_)]^2+^ (pH 5–6.5) and [Cu­(LH)]^+^ (pH 5.5–7.5). The first species formed starting from acidic pH is [Cu­(LH_2_)]^2+^ with Cu­(II) coordinated in the equatorial plane by two imidazole nitrogen atoms and two water molecules (observed expected λ_max_ = 650 nm; expected λ_max_ = 650 nm).^22,23^ By increasing the pH, a new species appeared with a maximum at 608 nm, corresponding to the maximum formation of [Cu­(LH)]^+^ (expected λ_max_ = 608 nm).^22,23^ This absorption is accounted for by the presence of two imidazole nitrogen and one peptide nitrogen atom. The last equatorial coordination position is completed by a water molecule. A schematic representation of the proposed coordination modes is shown in Figure 4.

Further proof of these coordination hypotheses was sought by spectrophotometric titrations of Pep1 or Pep2 with Cu­(II) at a HEPES buffered pH 7.4 (see below).

### Copper­(II/I)/Peptides Binding

The binding of Cu­(II) to the peptides was studied by UV–vis absorption and CD titrations of PepX with the metal at pH 7.4 (50 mM HEPES buffer). As shown in Figure S3 for Pep1 and Pep2, an absorption band appeared at ca. 585 nm, which is indeed characteristic of Cu­(II) bound in a (N_im_, 2x N^–^, O) equatorial coordination environment. Unfortunately, a marked drift in the baseline was observed above a Cu/Pep ratio of 1:1, ascribed to the precipitation or aggregation phenomena (Figure S3). The CD spectra present 2 bands at ca. 525 and 655 nm up to a 1:1.5 metal/peptide molar ratio. Beyond this ratio, CD data collection was hindered by the appearance of precipitates.

The affinities of Pep1 and Pep2 for Cu­(II) were calculated using spectrofluorimetric titration data (Figure 5A–D). As shown, the quenching of fluorescence emission of the tryptophan residue at 350 nm reaches an endpoint when 1 equiv of Cu­(II) was added, consistent with the formation of a 1:1 Cu­(II):Pep complex. The intensity of Trp emission as a function of the equivalents of copper­(II) added was treated using the HypSpec2014 software, using a 1:1 Cu­(II):peptide model. Data treatment provided log *K* values of 7.16(1) and 7.53(2) for Pep1 and Pep2, respectively (Table 2). The copper­(II) affinity for PepCtrl could not be determined because the change in the intensity was attributed to the dilution effects only (Figure S4). Overall, these data are in agreement with the potentiometric observations and indicate that at neutral pH, only one Cu­(II) is bound at the bis-histidine site.

**Figure 5:**
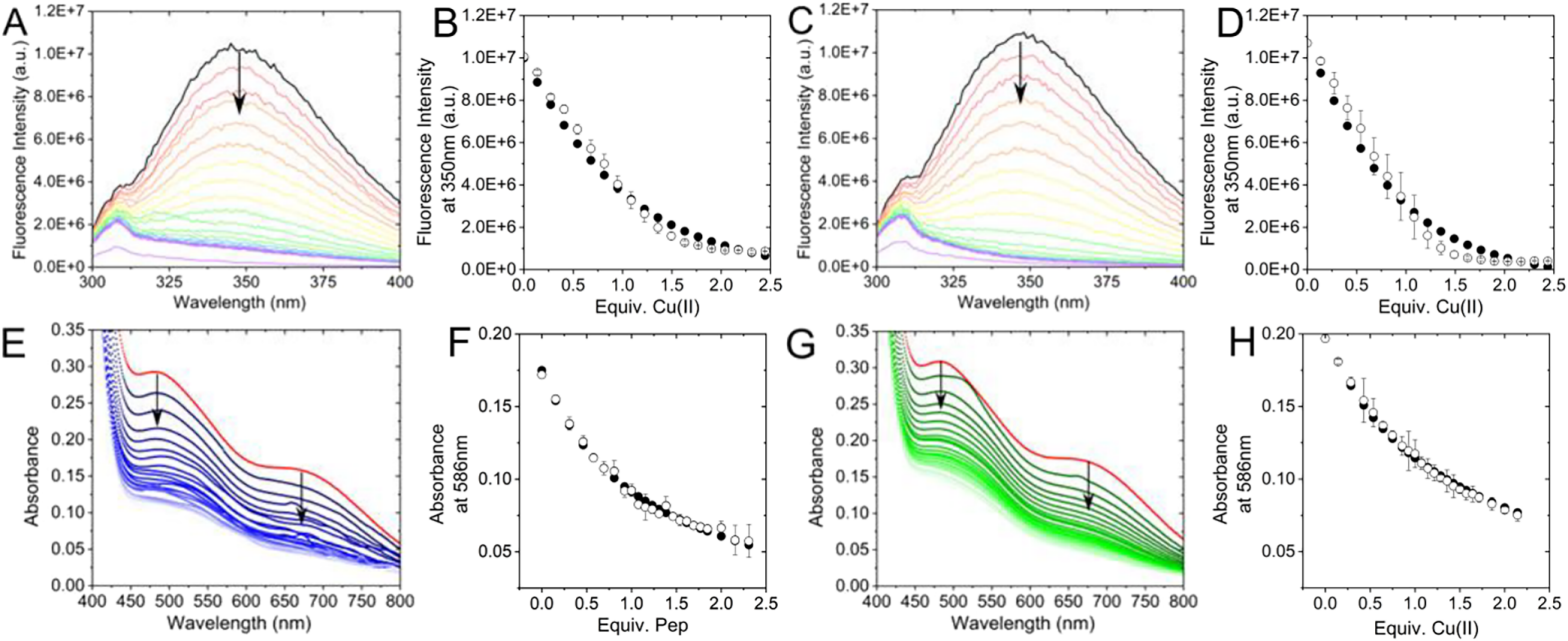
Top line: Representative fluorescence emission spectra for the titration of a solution of Pep1 (A) or Pep2 (C) with up to 2.5 equiv of Cu­(II) ([PepX] = 10 μM, 50 mM HEPES, pH 7.4). Cu­(II): PepX = 0 (black spectrum) to 2.5 (purple spectrum), with 0.1 equiv. additions. Fluorescence fittings at 350 nm are shown in panels (B) (Pep1) and (D) (Pep2). The observed absorbance is reported as open circles and calculated as filled circles. Bottom line: Representative UV–vis titration of [Cu^I^(CH3CN)­4]­BF4 and a metallochromic indicator (Fz) with Pep1 (E) and Pep2 (G). (Cu­(I):Fz = 1:2, [CuI] = 0.05 mM, 50 mM HEPES, pH 7.4). Cu­(I): PepX = 0 (red spectrum) to 2.3, with 0.1 equiv. additions. Absorbance fittings at 600 nm are reported in panel (F) (Pep1) and panel (H) (Pep2). The observed absorbance is reported as open circles and the calculated ones as filled circles. Error bars represent the average values from at least two independent replicates.

The conditional binding affinities of Pep1 and Pep2 for Cu­(I) were determined by UV–vis titration in the presence of the ferrozine (Fz) metallochromic indicator as a competing ligand. Figure 5E–H shows the spectra of a solution of [Cu^I^(Fz)_2_]^3–^ titrated with PepX at pH 7.4. Both absorption bands of the Cu­(I)/Fz adduct at 475 and 600 nm decrease upon the addition of the peptide, indicating that ferrozine is displaced from the copper coordination sphere with the formation of a colorless Cu­(I)/peptide adduct. The absorbance data in the range 450–700 nm as a function of the equivalents of peptide added were treated using a 1:1 Cu­(I):peptide model and using the formation constant of [Cu^I^(Fz)_2_]^3–^ as a fixed parameter (log β_[CuFz2]_^3–^ = 11.6).^22,23^ Data treatment yielded log *K* values of 8.98(2) and 9.8(1) for Cu­(I)­Pep1 and Cu­(I)­Pep2, respectively (Table 2). As for PepCtrl, the decrease in absorbance in the competition titration of the [Cu^I^(Fz)_2_]^3–^ solution is negligible and fully ascribable to dilution effects. This observation indicates that the affinity of PepCtrl for Cu­(I) is extremely low and cannot be determined from these data. Indeed, the His–His tandem site is known to be a binding site for both Cu­(I) and Cu­(II), and therefore, it is not surprising that PepCtrl does not show a significant affinity for both cations.

*E*′_0_^Cu(II/I)^, the reduction potential of free Cu^2+^ in aqueous solution, is +0.26 mV (vs NHE).^24,25^ With the affinity values of Pep1 and Pep2 for Cu­(I) and Cu­(II), we could calculate the reduction potential for the Cu­(II/I) couple in its adduct with the peptides. The values of these reduction potentials were around 380 mV vs NHE, as reported in Table 3.

**Table 3:** Logarithms of the Conditional Formation Constants (log *K*) of the Adducts of Pep1 and Pep2 with Cu­(II) and Cu­(I) at pH 7.4 (50 mM HEPES)^a^

	log *K*		*E*_0_ (mV)
	Cu(II)	Cu(I)	
Pep1	7.16(1)	8.98(2)	368(1)
Pep2	7.53(2)	9.8(1)	394(1)

a Reduction potentials (mV vs NHE) for the reduction of the Cu­(II) adducts with the Pep1 and Pep2 peptides are also reported. Standard deviations are given in parentheses.

It has been previously demonstrated that the presence of a tandem His–His binding site in a peptide promotes the reduction of Cu­(II) to Cu­(I).^16,26–29^ This behavior was previously observed in Aβ, αSyn, and tau peptide fragments, and was associated with a much higher affinity for Cu­(I) of bis-His sites compared to that of mono-His sites.^16,26–29^ Interestingly, the reduction potential of the R3 model peptide for the region of tau comprising the (His)_2_ site was +110 mV,^16^ which was much lower than that observed for the Cu-tau protein adduct (340 mV vs NHE).^30^ Here, the reduction potentials of the adducts of Cu­(II) with PepX are much closer to the value determined for the copper adducts with the full-length tau protein. Quite interestingly, this behavior makes unexpectedly PepX a better redox model for the tau protein than the R3 fragment.

### Oxidation of 4-Methylcatechol (4MC) and L/D-Dopa by Cu–PepX Complexes

Literature data show that copper­(II) bound at a His–His site in model peptides (e.g., prion protein or Tau) is capable of promoting the oxidation of catechols into quinones. For this process, a reaction mechanism was proposed for prion protein models.^31^ In this study, we attempted to demonstrate that Cu­(II) adducts with our peptides also exhibit pseudocatecholase activity. To this end, we used 4-methylcatechol (4-MC) or DOPA as substrates. Also, we tried to assess whether our peptides are capable of chiral discrimination of D- or L-DOPA, possibly resulting in a different rate of oxidation of the two enantiomers of the substrate.

Figure 6 shows an increase of absorbance at 401 nm in a system containing Cu­(II), Pep1 or Pep2, and 4-MC (blue and green dots, respectively). The increase of absorbance is due to the appearance of 4-methylquinone (4-MQ) as the product of oxidation in the presence of dioxygen. The kinetic profiles (absorbance vs time, Figure 6) show that the autooxidation of 4-MC (i.e., in the absence of Cu­(II) and peptides, pale gray dots) under the conditions of temperature, pH, and buffer used is a very slow process. In the presence of naked Cu­(II), the process becomes faster (dark gray dots). Further acceleration was observed when an equimolar amount of peptide (either Pep1 or Pep2) was present in the solution.

**Figure 6:**
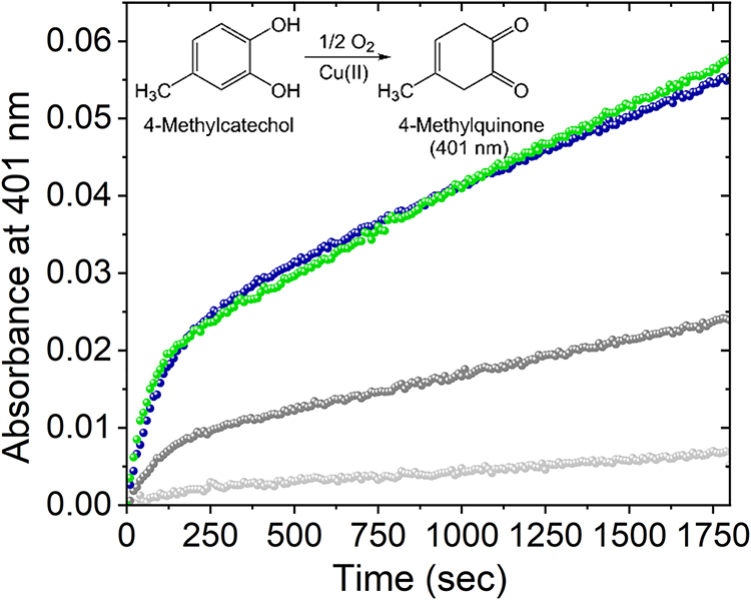
UV–vis kinetics of the oxidation of 4MC with Pep1 (blue) and Pep2 (green), and control in light gray 4MC alone and in the darker gray 4MC with copper (50 mM HEPES, pH 7.4, PepX 25 μM, Cu­(II) 25 μM, 4MC 3 mM).

The collection of spectra was limited to 1800 s (30 min) after triggering the reaction by the addition of the metal to the 4-MC solution. It has been previously reported^18,31^ that for longer reaction times, a change in the shape of the spectra was observed, indicating that 4-MQ reacts with the excess of 4-MC, forming a subproduct that possesses an absorption maximum at 480 nm and interferes with the measurement of the absorption of 4-MQ.^18,31^ Indeed, we have observed this behavior for our system, and therefore, we limited our absorbance data analysis to the 30 min time frame. Finally, it is important to highlight that the kinetic profile in the presence of the peptides is clearly biphasic, with an initial fast phase in the first 100 s, followed by a slower linear phase. This kinetic profile, similar to that previously reported for the oxidation of catechols by Cu-Amyloidβ,^29^ Cu-αSynuclein,^18,32^ and Cu-Tau^16^ is discussed in the DiscussionSection.

To gain information on the potential catalytic role of copper–Pep1 and copper–Pep2 complexes in oxidative reactions, we performed a comparative study of their oxidative activity against a catecholic substrate (4MC) by increasing the peptide concentration, the copper­(II) concentration, or varying the substrate concentration. The absorbance vs time plots grouped by the Cu­(II)/peptide ratio (Figure S5) show that the kinetic profiles are remarkably similar across Pep1 and Pep2 when the Cu­(II)/PepX ratio is the same. Therefore, these data confirm that the catalysis is copper-promoted, as an increase of the total copper concentration in the system reflects a corresponding increase of the slope of the catalytic phase. Similarly, no significant differences are observed between the catalytic phase at Cu­(II)/Pep ratios of 1:2 and 1:4 when we work at fixed Cu­(II) concentration with increasing amounts of peptide (Figure S6). This is due to the higher percentage of Cu­(II) bound to the peptide, which amounts to ca. 95% of the total Cu­(II), considering the log *K* and 25 μM. Thus, the increase of peptide concentration should have a very limited effect on the percentage of bound copper and, therefore, on the catalytic effect.

## Discussion

In this work, we first examined the speciation of the Cu­(II)–PepX systems, where the two peptides Pep1 and Pep2 bear a His–His tandem in their sequence. The species formed in the pH range 3.5–11.5 are all monometallic species with a 1:1 copper:peptide ratio. The data obtained by potentiometric and spectrophotometric titration revealed that the two His residues are involved in Cu­(II) binding at pH above 5 (Figures 2 and 4). In the pH range 5.0–6.5, the major species is [Cu­(LH_2_)]^2+^, which possesses an absorption maximum at 650 nm (Figure 3). For this complex, we advance the coordination of two imidazole groups on the equatorial plane of Cu­(II) with the formation of a macrochelate and two water molecules. Indeed, for this (2 × Im, 2 × H_2_O) equatorial coordination environment, the expected absorption maximum is at 650 nm, which is fully consistent with the experimental one. Moving to higher pH values, in the 5.5–7.5 range, the major species is [Cu­(LH)]^+^, which possesses an absorption maximum at 608 nm (Figure 3). This absorption maximum is fully consistent with the equatorial arrangement of two imidazole and one deprotonated peptidic nitrogen, as shown in Figure 4.

Above pH 6.7, the major species is [CuL]: at pH 7.4, this species accounts for 72 and 79% of the total copper for Pep1 and Pep2, respectively. In this species, the equatorial coordination positions of Cu­(II) are occupied by one imidazole nitrogen, two deprotonated peptide nitrogen atoms, and one water molecule, as suggested by the spectroscopic data analysis. As for the second imidazole function (see Figure 1), the maximum absorption of [CuL] at 584 nm is compatible with both the coordination of this group at the axial position of Cu­(II) or its presence as a noncoordinated group. In this respect, we may suggest the hypothesis of an equilibrium between coordinated and uncoordinated axial imidazole, but no further insights can be acquired using the current data.

The values of the global formation constants reported in Table 3 allowed the calculation of the formation log *K* of the copper­(II) adducts with the peptides using the HySS software. These values are 7.16 and 7.53 for Pep1 and Pep2, respectively, and are fully consistent with the value determined for the model peptides of the R3 tau fragment (*K*_d_ = 71 nm, log *K* = 7.1).^16^ As for Cu­(I), the spectroscopic titration data of the peptides with Cu­(II) at pH 7.4 indicate the formation of solely 1:1 Cu:peptide adducts at this pH, with formation log *K* for the adducts well in line with those previously determined for the model peptides of the R3 tau fragment (log *K* = 8.98 and 9.8 for Pep1 and Pep2, respectively, and 10.1 for R3 ^16^).

The two peptides differ only by an alanine residue placed in Pep2 immediately downstream of the His–His site. The log *K* values for the binding of both Cu­(I) and Cu­(II) reveal that the affinity of Pep2 for copper in both oxidation states is slightly higher than that of Pep1. Interestingly, a consequence of this increase in the stability of the copper adducts compared to those of Pep1 is that the *E*_0_ for the Cu­(I/II) couple is not significantly different for the two peptides. Taken together, and as could reasonably be expected, all the experimental information suggests that the presence of the alanine residue after the His–His site does not reflect differences in the coordination features of the Cu­(His)_2_ site but still impacts the stability of the adducts. We may predict that this may be due to a change in the acidity of the amidic NH groups, or to some second-shell interactions between the Gln residue and the His–His site, although current data cannot discriminate between these effects.

Overall, our data on the stability of Cu­(I) and Cu­(II) adducts with the peptides and the coordination environment of Cu­(II) in its complexes are consistent with those observed for other model peptides bearing a His–His tandem site.^16,18,29,32,33^ In particular, as put forward for copper adducts with tau fragments, it is possible to hypothesize that for Pep1 and Pep2, the Cu­(II) species relevant to the pseudocatecholasic redox activity is also [CuLH]. Indeed, in these species, copper­(II) is (N^–^, 2x N_im_, O_water_) coordinated, with two imidazole groups on the equatorial plane. The hypothesis is that this coordination mode permits Cu­(II) to be easily reduced to Cu­(I), which is likely coordinated to the two His in a linear fashion.^29,34–36^ Interestingly, this species accounts for only ca. 75% of the total copper at pH 7.4. Nevertheless, the proposed mechanism of oxidation of catechols in the literature for these Cu­(His)_2_ sites indicates that copper is rapidly reduced to Cu­(I), which may become the predominant oxidation state in the first phase of the catalytic cycle.

The kinetic profiles for the oxidation of 4-MC to 4-MQ (Figure 6) are fully consistent with those reported for the Cu/R3 peptide fragments of Tau.^16^ We therefore demonstrate that the oxidation of 4-MC follows the same mechanism of reaction, which is schematically shown in Figure 7. The initial phase in the kinetic profiles lasts until 100 s from the beginning of the reaction; this phase is associated with a higher rate of reaction compared to that observed after ca. 250 s (linear phase). The mechanistic model associates with the initial phase is the rapid reduction of Cu­(II)/peptide to Cu­(I)/peptide operated by the substrate, and it is referred to as the *stoichiometric phase*. During this phase, 4-MC is transformed to 4-methylsemiquinone (Figure 7), which in turn rapidly dismutates into 4-MC and 4-MQ, with the appearance of the absorption band of the latter at 401 nm. Cu­(I)/peptide subsequently binds to a second 4-MC molecule, followed by dioxygen. The 4 electrons required for O_2_ to be reduced to water are provided by the bound 4-MC molecule, Cu­(I), and a second 4-MC substrate that eventually provides the last electron to yield 4-methylsemiquinone. Overall, the process produces two 4-MQ molecules, which are indeed responsible for the increase in the absorption band at 401 nm. The binding of dioxygen, from previous studies, is expected to be the rate-determining step of the process, ultimately accounting for the *linear (catalytic) phase* of the kinetic profile.^31^

**Figure 7:**
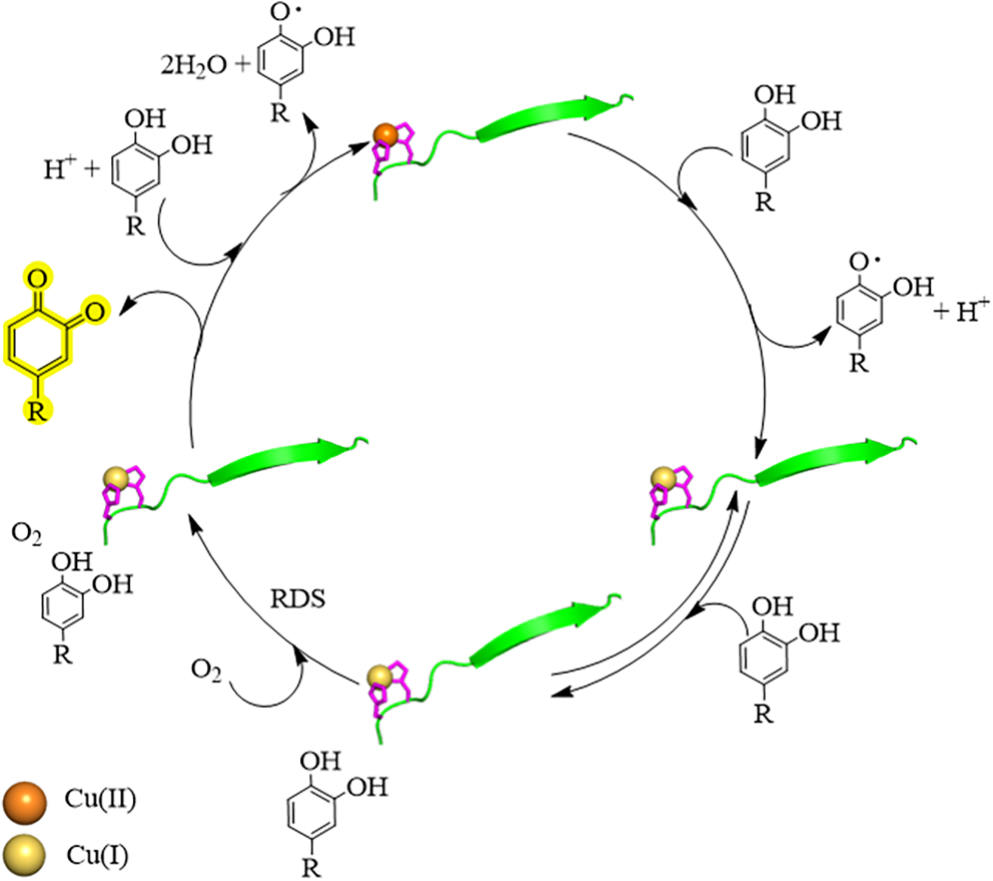
Schematic representation of the mechanism of catechol oxidation. The peptide is shown in green, the bis-histidine site in pink, Cu­(II) in orange, and Cu­(I) in yellow.

Kinetic analysis of the rate-determining step as a function of 4MC concentration and the derived catalytic parameters are shown in Figure S8 and Table S1. The turnover number (*k*_cat_), *K*_M_, and catalytic efficiency (*k*_cat_/*K*_M_) of the two peptides do not differ significantly (Table S1), confirming the comparable behavior of the metal/PepX adducts in promoting 4MC oxidation.

Both peptides bear the same His–His site with an identical Ac-GW N-terminus. The differences between the two sequences lie, therefore, only in the presence of the small Ala residue in Pep2 as a spacer between HH and Gln. As expected, the presence or absence of the Ala spacer did not affect the oxidation rate of 4-MC, which is also a substrate of relatively small dimensions and does not present charged or polar groups other than the two OH that may interact with the polypeptide chain.

Within the constraints of molecular mechanics approximations, even if steric hindrance in the immediate vicinity of the copper center appears to be limited, chiral recognition is predominantly dictated by second-shell interactions (i.e., hydrogen bonds) and not only by steric factors. Nevertheless, given the chirality of the peptide scaffold, a certain degree of stereodiscrimination could be expected. To probe chiral discrimination, we employed D- and L-Dopa as the substrates. For these isomers, second-shell effects such as hydrogen bonds between the charged groups of Dopa and the polypeptide chain could play a significant role in chiral recognition. However, the rates of oxidation of D- and L-Dopa are not significantly different.

## Conclusions

In this study, we examined the stability of Cu­(I) and Cu­(II) complexes of SpyTag peptides bearing a His–His tandem site at the N-terminus. Overall, our results demonstrate that the presence of an alanine spacer between the (His)_2_ site and the glutamine residue just upstream of the SpyTag IVMVD sequence increases the stability of both Cu­(I) and Cu­(II) adducts. Quite interestingly, this increase in stability for both oxidation states compensated for each other and did not significantly impact the value of the reduction potential of the Cu­(II)/Pep2 adduct compared to that of Pep1.

The Cu­(II)/peptide adducts were demonstrated to promote the oxidation of catechols into quinones. On this ground, however, we were not able to observe any significant differences in the kinetic profiles of the L- and D-isomers of Dopa. The fluxionality of these unstructured peptides is likely the origin of this lack of enantioselectivity. Nevertheless, it will be interesting in follow-up studies to examine the possible presence of stereoselectivity for these copper-binding sites present on reconstituted Spy proteins.

## Experimental Section

The peptides were purchased from Genescript with a purity of ≥95%. All other chemicals were purchased from Sigma-Aldrich.

### Stock Solutions

Concentrated (ca. 0.5–2 mM) solutions of Pep1, Pep2, and PepCtrl were prepared by weight in 50 mM HEPES buffer at pH 7.4. The peptide concentration was determined by UV–vis spectroscopy (ε_Trp,280nm_ = 5690 M^–1^ cm^–1^).^37,38^ CuCl_2_ stock solutions were prepared by weight from analytical-grade metal salts and standardized by complexometric titrations with EDTA following standard protocols.^39^ Aqueous HEPES buffer solutions (50 mM, pH 7.4) were prepared in doubly distilled water.

### UV–Vis Spectroscopy

UV–vis spectra were collected at 298.2 K on a Cary 3500 spectrophotometer using 1 cm path length quartz anaerobic cuvettes. Tetrakis­(acetonitrile) copper­(I) tetrafluoroborate [Cu^I^(MeCN)_4_]­(BF_4_) was kept under an inert atmosphere until use. All solutions were prepared and handled in an inert atmosphere glovebox to prevent Cu­(I) oxidation. A copper­(I) stock solution (1 mM) was prepared in acetonitrile. The Cu­(I) concentrations were determined using BCS.^40–42^ Competitive titration was used to determine the affinity of Cu­(I) to peptides. The titration of the [Cu^+^(Fz)_2_]^3–^ adduct was carried out by quenching the absorbance of the complex^26^ by titrating with peptides in dimethyl sulfoxide (DMSO). Stock solutions (1 mM) of the peptide were prepared by weight in DMSO under anaerobic conditions. The maximum amount of DMSO in the sample during titration never exceeds 10% of the total volume. Under these experimental conditions, the effect of DMSO on the determination of log β of formation constants of copper complexes can be considered negligible.^43^

A solution of 0.05 mM Cu­(I), 10 mM ascorbic acid, 10 mM hydroxylamine chloride, and 0.16 mM Fz (3-(2-pyridyl)-5,6-diphenyl-1,2,4-triazine-4′,4″-disulfonic acid sodium salt) in 50 mM HEPES buffer, pH 7.4, was prepared in a 1 cm quartz anaerobic cuvette in an inert atmosphere glovebox and titrated with 1 mM peptides in DMSO (Fz:Cu­(I) = 2:1, Cu:Pep = 1:0–2.3). Hydroxylamine hydrochloride and ascorbic acid were used as sacrificial reductants to prevent Cu­(I) oxidation^41,42^ during peptide titration.

### pH Titration

UV–vis spectra were collected at 298.2 K on a Cary 3500 spectrophotometer, equipped with a Peltier thermostat, using a 1 cm path length quartz cuvette. A solution was titrated over a pH range of 3–11 at 25 °C by using a carbonate-free potassium hydroxide (KOH) solution (0.0636 M) as the titrant. The ionic strength was adjusted to 0.1 M with KCl. The ligand (peptide) concentration was 0.3 mM, and the metal-to-ligand ratio was 1:1.2. pH titration was performed using an Omnis automatic titrator system (Metrohm, Herisau, Switzerland) with a glass electrode.

### Fluorescence Spectroscopy

Fluorescence emission spectra were recorded at 298.2 K using a Horiba Jobin Yvon Fluoromax-3 spectrofluorometer with quartz cuvettes of 1 cm path length and a fixed excitation wavelength of 280 nm (slit width = 2 nm), corresponding to the band of absorption of Trp. Samples of Pep1, Pep2, or PepCtrl were prepared in 50 mM HEPES solution at pH 7.4 to obtain a final protein concentration of 0.5 mM. Spectrophotometric titrations were carried out by adding CuCl_2_ 0.587 mM to sample solutions up to 5 metal equiv. with respect to the peptide. All titrations were performed in duplicate. The Cu­(II) binding constants were determined by tryptophan fluorescence quenching for all peptides. Spectral data were treated using the software HypSpec2014.^2,44^

### Circular Dichroism (CD) Spectroscopy

CD spectra were collected using a Jasco J-1500 CD spectrometer equipped with a Peltier thermostat at 298.2 K, with quartz cuvettes of 1 cm path length. Samples of Pep1, Pep2, or PepCtrl were prepared in 50 mM HEPES solution at pH 7.4, with a final peptide concentration of 0.4 mM. Spectrophotometric titrations were carried out by adding Cu­(II) to the sample solutions up to 1.5 equiv. All titrations were performed in duplicate.

### Potentiometric Measurements

Stability constants of the proton as well as Cu­(II) complexes of the peptides were calculated from the pH-metric titration curves obtained under a nitrogen atmosphere (in order to avoid the appearance of carbonate in the sample) over the pH range 3.5–11.5 at 298.2 K and an ionic strength of 0.1 M KCl using a total volume of 1.5 mL. Potentiometric titrations were carried out using an automatic Metrohm OMNIS titrator equipped with a Metrohm semimicro analogic glass electrode. The system was controlled by a PC to monitor the attainment of equilibrium through pH measurements vs time and the addition of titrant aliquots. The thermostabilized glass cell was equipped with a magnetic stirring system, a microburet delivery tube, and an inlet–outlet tube for nitrogen. The solutions were titrated with 0.0583 M carbonate-free KOH, which was previously standardized against potassium hydrogen phthalate.^45^ The electrodes were calibrated daily for hydrogen ion concentration by titrating HCl with KOH under the same experimental conditions as those mentioned above. The Gran method^45^ allowed us to confirm the purities and concentrations of the ligand (Pep1, Pep2, or PepCtrl) solutions (checked by UV–vis spectroscopy). The peptide concentration was 0.15 mM, and the Cu­(II) to ligand molar ratio was 1:1.2. Stability constant calculations were performed using the HyperQuad 2013 program.^2^ Standard deviations were calculated by HyperQuad 2013 and refer to random errors only. The distribution and competition diagrams were computed with the HySS program.^46^

### Kinetics of the Oxidation of Catecholic Substrate 4-Methylcatechol (4-MC) Oxidation

The catalytic oxidation of 4-MC by Cu­(II) was studied at 25 °C in 50 mM HEPES buffer at pH 7.4, saturated with atmospheric oxygen. The reaction was monitored by UV–vis spectroscopy following the absorption band of the chinone at 401 nm (ε = 1550 M^–1^ cm^–1^).^18^ The effects of Pep1, Pep2, or PepCtrl peptides on the copper-catalyzed 4-MC oxidation were evaluated. All measurements were performed in duplicate.

The effect of peptide or copper concentration was investigated by keeping the amount of copper or peptide (25 μM), respectively, at a constant substrate concentration (3 mM).

The effect of substrate concentration was investigated by keeping the amounts of Cu­(II) (25 μM) and PepX (25 μM) constant and varying the MC concentration from 0.3 to 4.0 mM.

### Dopa Oxidation

Two enantiomers, L-DOPA and D-DOPA: The catalytic oxidation of the DOPA (3,4-dihydroxy-L/D-phenylalanine) substrate by Cu­(II) was investigated at 20 °C in 50 mM HEPES at pH 7.4, saturated with atmospheric oxygen. The oxidation of both enantiomers L- and D-Dopa was evaluated. The reaction was monitored by UV–vis spectroscopy following the absorption band of chinone at 470 nm. The effects of Pep1, Pep2, or PepCtrl peptides on copper-catalyzed DOPA oxidation were evaluated. All measurements were performed in duplicate.

## Supplementary Material



## Notes

Peptides protonation; CD for folding of peptides; UV–vis titration at pH 7.4 for Cu­(II)­Pep1 and Cu­(II)­Pep2; determination of Cu­(II) and Cu­(I) binding affinities to PepCtrl; 4MC oxidation profiles by varying the Cu­(II):peptide ratio or substrate concentration; and L-/D-Dopa oxidation kinetic profiles (PDF)
