# Supplementary material for: Stability and Pseudocatecholase Activity of Artificial Bis-Histidyl Copper Peptides
Source: Inorg Chem. 2025 Oct 8;64(41):20567–76. doi: 10.1021/acs.inorgchem.5c02080 (PMC12541693; doi:10.1021/acs.inorgchem.5c02080)
Supplement: Supplementary file 1 [file ic5c02080_si_001.pdf]

# SUPPORTING INFORMATION for

# Stability and Pseudocathecolase Activity of Artificial Bis- Histidyl Copper Peptide.

Chiara Bottoni, Matteo Tegoni, Valentina Borghesani*

Department of Chemistry, Life Sciences, and Environmental Sustainability, University of Parma, Parco Area delle Scienze 17A, 43124 Parma, Italy
E-mail: valentina.borghesani@unipr.it

**Contents**

**Binding constants: data treatment and calculations.**

**Figure S1.** Diagram distribution of protonation of (A) Pep1, (B) Pep2 and (C) PepCtrl.

**Scheme S1.** Scheme of deprotonation of PepX (X=1 or 2) ($[H_5L]^{3+}$).

**Figure S2.** Titration with copper(II) of Pep1, Pep2 and PepCtrl monitored by Circular Dichroism (CD) at pH 7.4

**Figure S3.** Titration with copper(II) of Pep1 and Pep2 monitored by UV-Visible at pH 7.4

**Figure S4** Titration of PepCtrl with Cu(II) monitored by fluorescence emission and titration of PepCtrl with Cu(I) monitored by UV-Visible by competition with metallochromic indicator (Fz) at pH 7.4

**Figure S5.** UV-Visible kinetics profiles of 4MC oxidation catalyzed by Cu-Pep1 and Cu-Pep2 at pH 7.4, at fixed peptide concentration and increasing Cu(II) concentration.

**Figure S6.** UV-Visible kinetics profiles of 4MC oxidation catalyzed by Cu-Pep1 and Cu-Pep2 at pH 7.4, at fixed Cu(II) concentration and increasing peptide concentration.

**Figure S7.** UV-Visible kinetics profiles of 4MC oxidation catalyzed by Cu-Pep1 and Cu-Pep2 at pH 7.4, at fixed peptide:Cu(II) 1.1 ratio and increasing 4MC concentration.

**Figure S8.** Initial oxidation rate of 4MC oxidation as function of substrate concentration (4MC) at fix 1:1 PepX:Cu(II) (PepX = Pep1 or Pep2) concentration at pH 7.4.

**Table S1.** Kinetic parameter for the catalytic oxidation of 4MC at pH 7.4.

**Figure S9.** UV-Visible kinetics profiles of L-/D- DOPA oxidation catalyzed by Cu-Pep1 and Cu-Pep2 at pH 7.4

**Figure S10.** Calculated UV-Visible spectra of Cu(II):Pep1 and Cu(II):Pep2, based on potentiometric complexation model

**Table S2.** Experimental and Calculated absorption of Cu(II) complexes of Pep1 and Pep2.

## Binding constants: data treatment and calculations.

All spectroscopic titration experiments were performed in duplicate, on independently prepared samples. The emission intensities recorded and the absorbances recorded in direct titration experiments (see. Figure 5 and S4) were treated as a full dataset in the range 300-400 and 400-800 nm, respectively.

Data treatment was carried out using the HypSpec 2014 software which allows to treat absorbance or emission at multiple wavelengths simultaneously, and to refine simultaneously multiple formation constants of metal complexes using a least-square regression procedure.[1,2] For each system studied, data from different titrations were treated together.

Affinities of Peptides for Cu(II) were studied by direct fluorescence titrations of PepX peptides (P = Pep1, Pep 2 and PepCtrl) with Cu(II). Best fit of the spectra dataset was obtained taking into account the formation of 1:1 copper/peptide adducts (charges omitted):

$$Cu + P = [Cu(P)]$$

The refined log $K_f$ values for this equilibrium are reported in Table 3.

The log $K_f$ of $Cu^{2+}$/HEPES adduct at pH 7.4 was taken into account in the calculations (log $K_f$ = 2.9, calculated using HySS from data reported in ref. [3]).

Affinities of peptide with Cu(I) were studied by competition UV-Visible titrations of Cu(I)/Ferrozine adduct with PepX (P = Pep1, Pep2 and PepCtrl). The spectra dataset were treated to obtain the $K_f$ of binding of Cu(I) to the bis-hystidine site.

The spectral dataset was treated similarly to that of the spectrofluorimetric titration of PepX with Cu(II), as reported above. Fit of the UV-Visible competition spectra dataset was obtained taking into account the formation of 1:2 Copper/ferrozine (Fz) and 1:1 copper/peptide adducts (charges omitted):

$$Cu + 2Fz = [Cu(Fz)_2]$$

$$Cu + P = [Cu(P)]$$

The logarithm of the first formation equilibrium was used as a fixed parameter (log $K$ = 11.6, ref. Alies et *al.*[4]). The refined log $K_f$ values for the second equilibrium are reported in Table 3.

All spectra datasets were treated using the speciation models reported above. Data treatment was carried out using the HypSpec2014 software that allowed to treat all the equilibria simultaneously. In all data treatment, the spectra dataset from independent experiments were treated together.

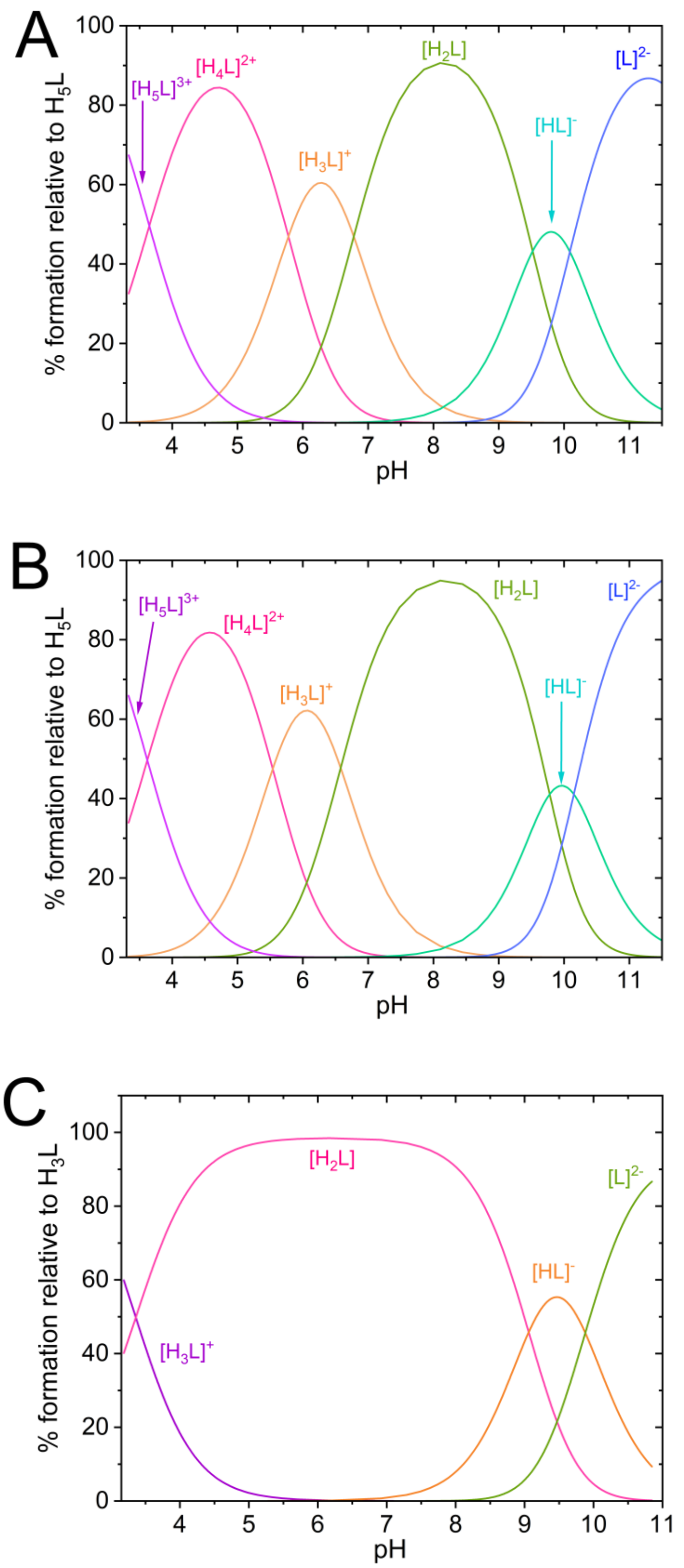


**Figure S1.** Diagram distribution of protonation of (A) Pep1, (B) Pep2 and (C) PepCtrl. $C_L$ = 0.3 mM, $T$ = 298.2 K, $I$ = 0.1 M (KCl).

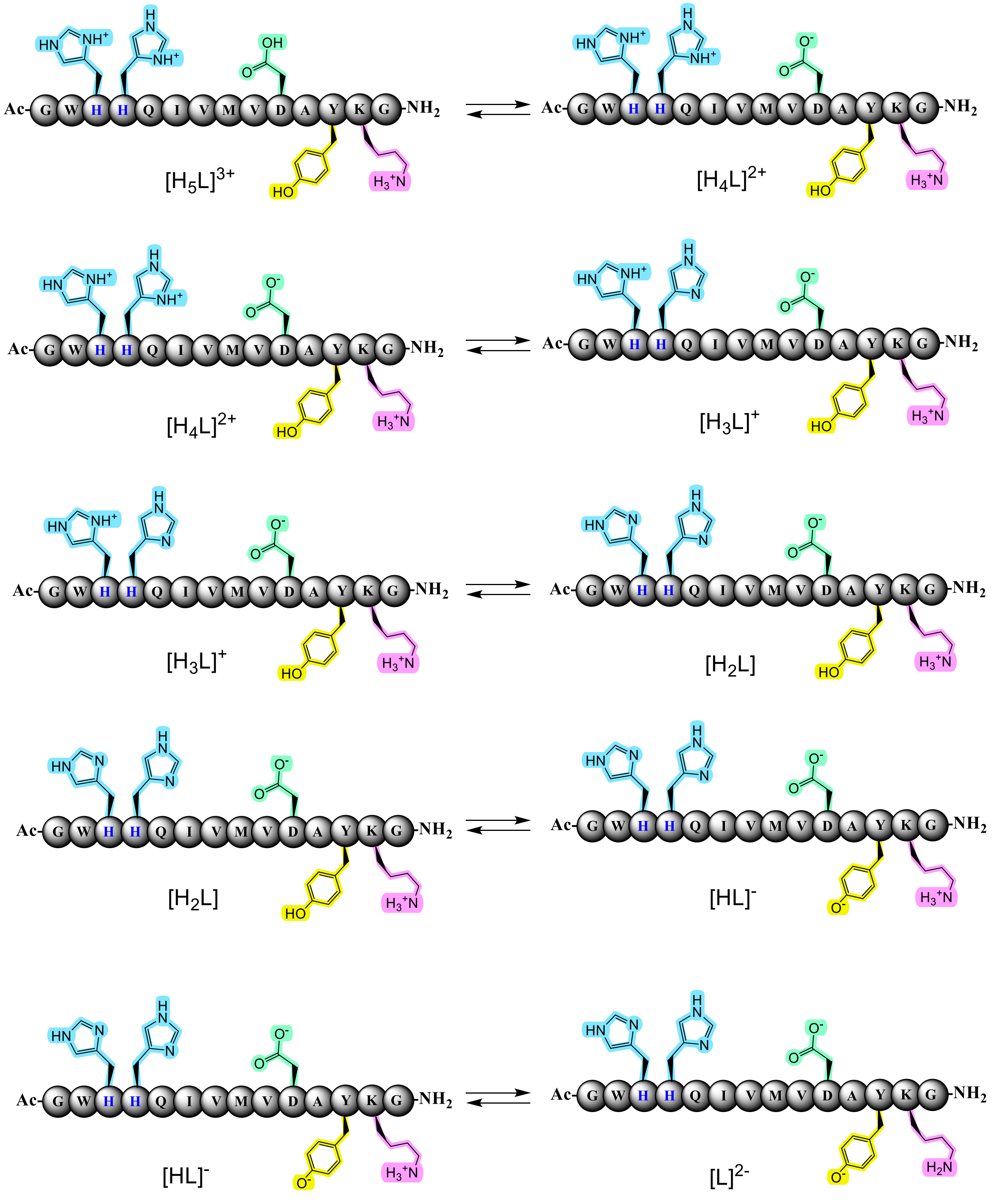


**Scheme S1.** Scheme of deprotonation of Pep1 ($[H_5L]^{3+}$). Pep2 behaves in the same way.

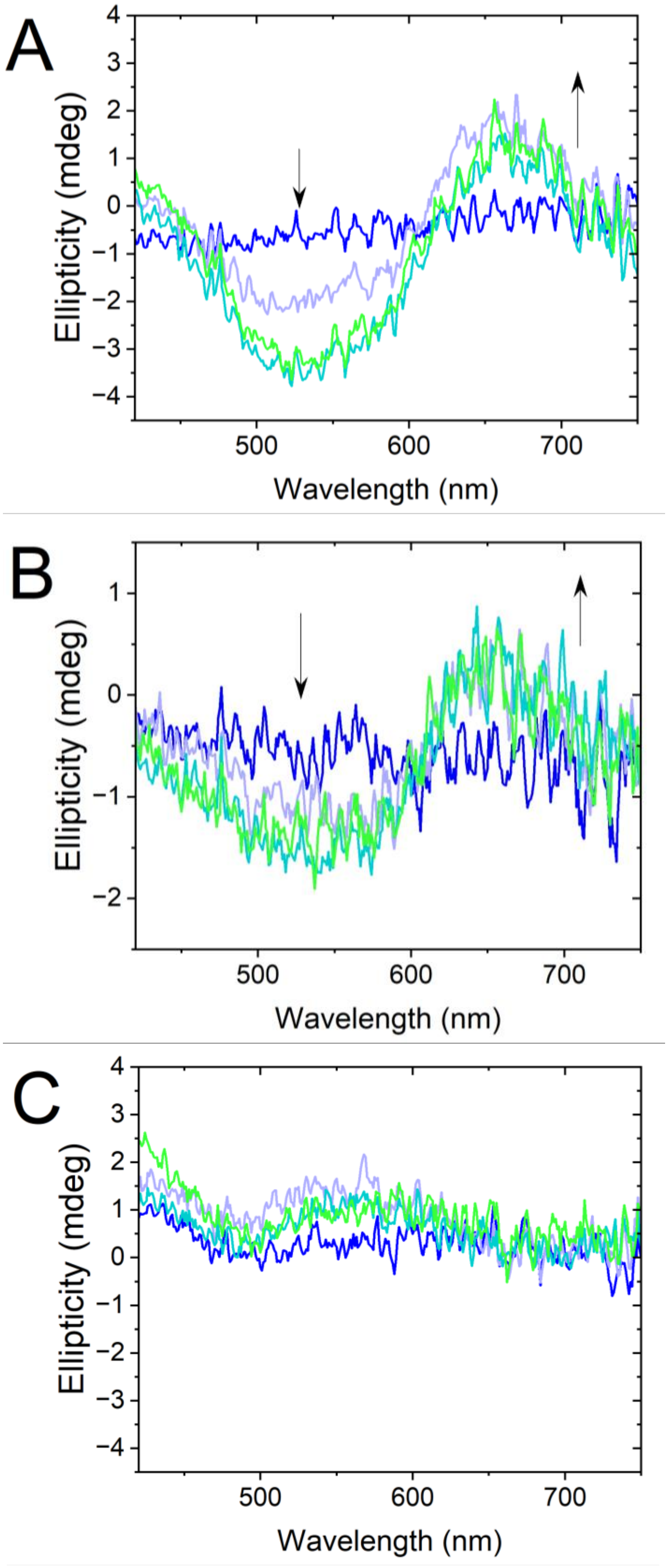


**Figure S2.** Folding for Pep1 (Panel A), Pep2 (Panel B) and PepCtrl (Panel C) titrated with Cu(II) monitored by circular dichroism at pH 7.4. [PepX] = 0.4 mM, [Cu(II)] = 0-0.6 µM. Buffer 50 mM HEPES pH 7.4, at 298 K. Apo peptide in blue, 0.5 equiv. of Cu(II) in light purple, 1.0 equiv. of Cu(II) in teal and 1.5 equiv. of Cu(II) in green.

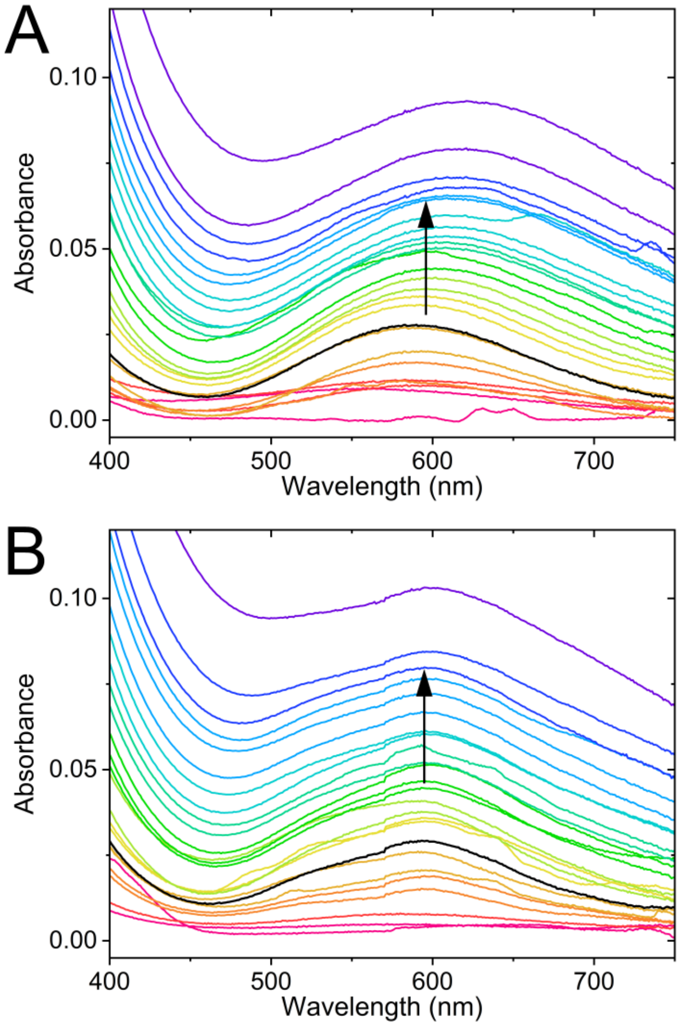


**Figure S3.** UV-Vis titration of $CuCl_2$ into a solution of 0.3 mM Pep1 (A) or Pep2 (B) at pH 7.4 in 50 mM HEPES buffer, $I$ = 0.1 M KCl. Peptide] = 0.3 mM, [Cu(II)] = 0-0.9 mM. Spectra at 1 equivalent of Cu(II) is depicted in black.

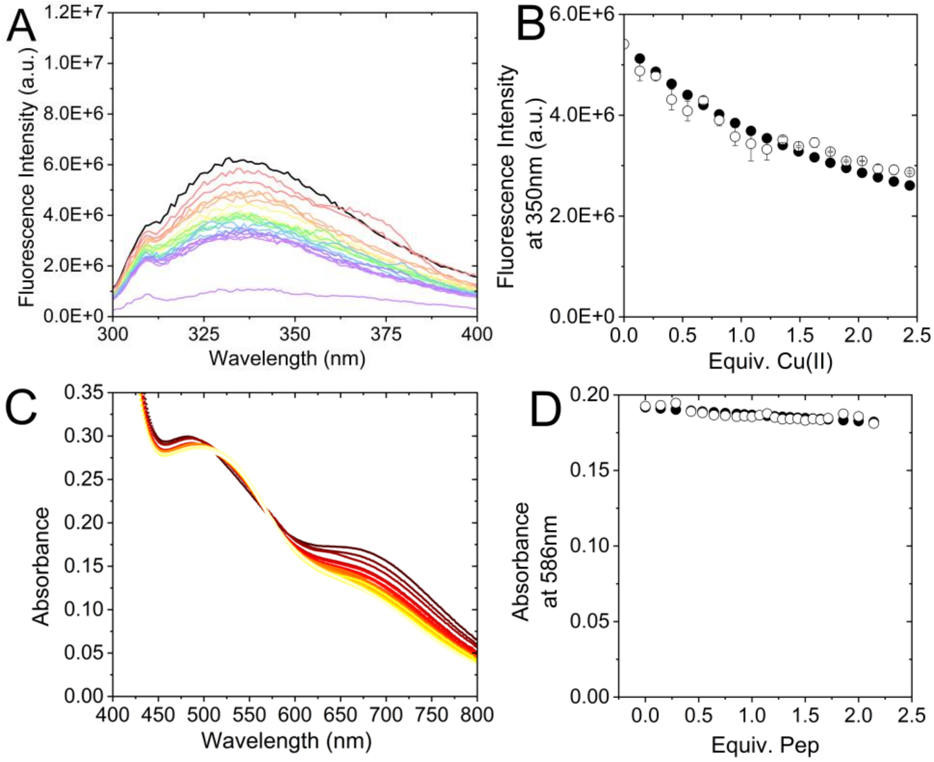


**Figure S4.** Representative fluorescence emission spectra (Panel A) for the titration of a solution of PepCtrl with up to 2.5 equiv. of Cu(II) ([PepX] = 10 μM, 50 mM HEPES, pH 7.4). Cu(II): PepX = 0 (black spectrum) to 2.5 (purple spectrum), with 0.1 equiv. additions. Fluorescence fitting at 350 nm are reported in Panel B (PepCtrl). Observed absorbance is reported as open circles and calculated one as filled circles. Representative UV-Visible titration of $[Cu(CH_3CN)_4]BF_4$ and a metallochromic indicator (Fz) with PepCtrl (Panel C) Cu(I):Fz = 1:2, $[Cu^+]$ = 0.05 mM, 50 mM HEPES, pH 7.4). Cu(I): PepX = 0 (yellow spectrum) to 2.3, with 0.1 equiv. additions. Absorbance fitting at 586 nm are reported in Panel D (PepCtrl). Observed absorbance is reported as open circles and calculated one as filled circles. Error bars result from averaging the values from at least two independent replicates.

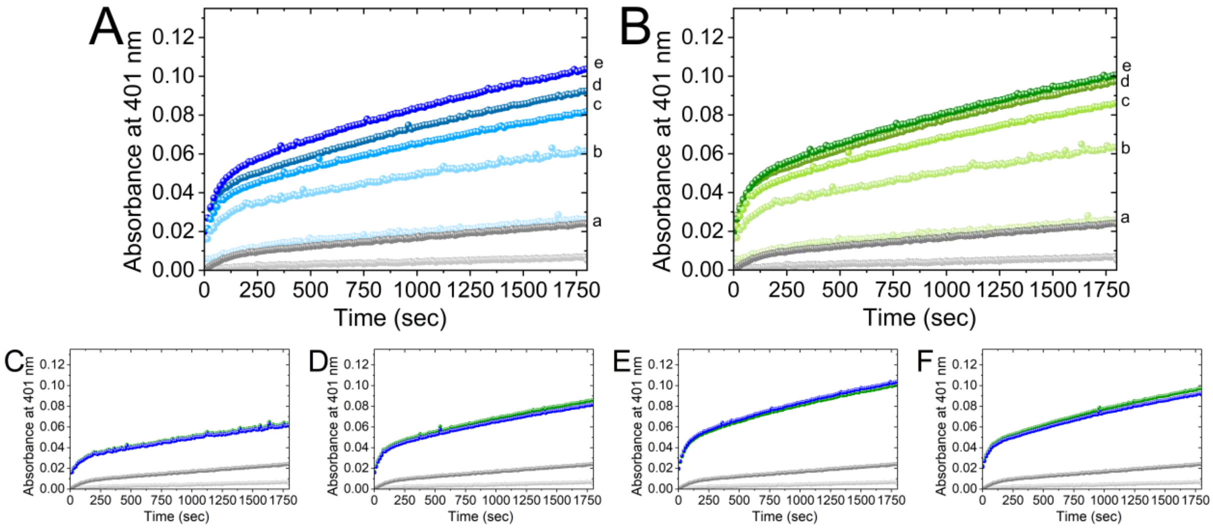


**Figure S5** Kinetic profiles for the oxidation of 4MC reported as absorbance at 401 nm recorded as a function of time.
**A-B**: kinetic profiles observed for Pep1 (A) and Pep2 (B) at fixed peptide concentration (25 µM) and increasing concentration of Cu(II). $C_{peptide}$ = 25 µM, [MC] = 3 mM, 50 mM HEPES pH 7.4, T = 293.2 K. $C_{Cu(II)}$ = 0 (a), 6.25 (b; 0.25 eq.), 12.5 (c; 0.5 eq.); 18.75 (d; 0.75 eq.) and 25 µM (e, 1 eq. *vs*. peptide). Increasing intensities in the shade of the same color (Pep1 in blue and Pep2 in green) indicate increasing copper(II) concentration. The kinetic profile for 4MC auto-oxidation is reported in light gray. The kinetic profile in the presence of 25 µM copper(II) in the absence of peptide is reported in dark gray, while the kinetic profile for 4MC auto-oxidation is reported in light gray.
**C-F:** kinetic profiles for the two different peptides grouped for the same peptide:copper(II) molar ratio 4:1 (C), 2:1 (D); 1.33:1 (E), 1:1 (F). Conditions correspond to those in profiles b, c, d and e of the upper row.

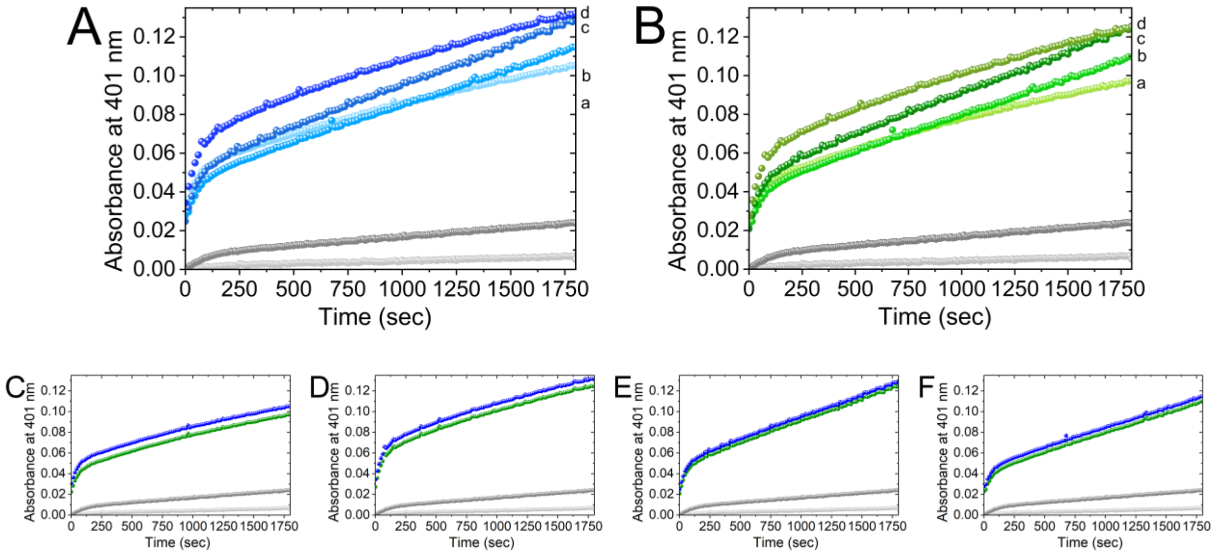


**Figure S6** Kinetic profiles for the oxidation of 4MC reported as absorbance at 401 nm recorded as a function of time.
**A-B:** kinetic profiles observed for Pep1 (C) and Pep2 (D) at fixed copper concentration (25 µM, profiles a-c) and increasing concentration of peptide. $C_{peptide}$ = 25 (a; 1 eq.), 50 (b; 2 eq.) and 100 µM (c; 4 eq. *vs* peptide). Increasing intensities in the shade of the same color (Pep1 in blue and Pep2 in green) indicate increasing peptide concentration. Kinetic profile d was collected with $C_{Cu(II)}$ = 50 µM and $C_{peptide}$ = 25 µM (Cu:peptide 2:1). [4MC] = 3 mM, 50 mM HEPES pH 7.4, T = 293.2 K. The kinetic profile for 4MC auto-oxidation is reported in light gray. The kinetic profile in the presence of 25 µM copper(II) in the absence of peptide is reported in dark gray, while the kinetic profile for 4MC auto-oxidation is reported in light gray.
**C-F:** kinetic profiles for the two different peptides grouped for the same peptide:copper(II) molar ratio 1:1 (C), 2:1 (D); 4:1 (E), 1:2 (F). Conditions correspond to those in profiles b, c, d and e of the upper row.

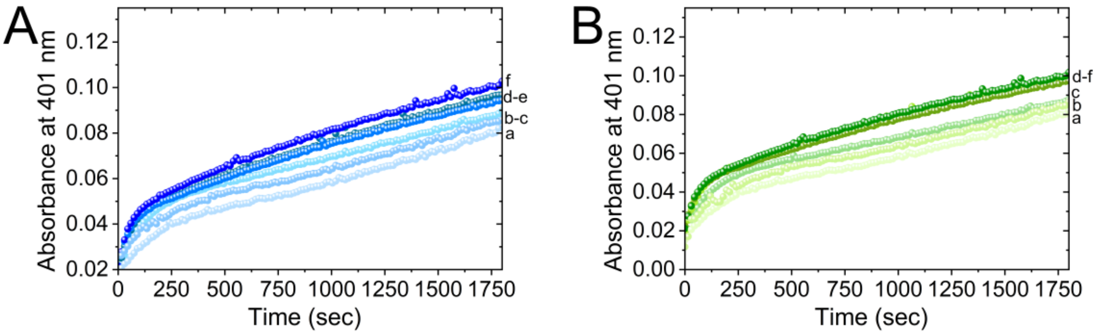


**Figure S7** Kinetic profiles for the oxidation of 4MC reported as absorbance at 401 nm recorded as a function of time for Pep1 (A) and Pep2 (B) at fixed peptide and copper concentration (25 μM each) and increasing concentration of 4MC substrate. $C_{4MC}$ =0.3 mM (a), 0.6 mM (b), 1mM (c), 2 mM (d) 3mM (e) and 4mM (f). Increasing intensities in the shade of the same color (Pep1 in blue and Pep2 in green) indicate increasing 4MC concentration. Kinetic profile was collected with 50 mM HEPES pH 7.4, T = 293.2 K.

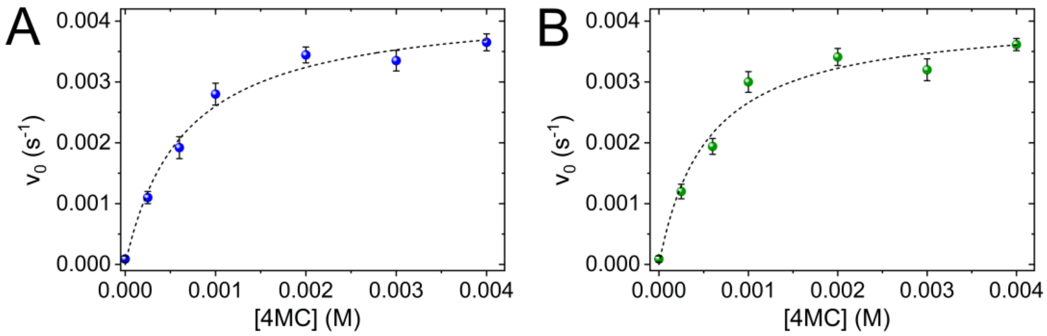


**Figure S8** Initial oxidation rates of 4MC in 50 mM HEPES pH 7.4 as function of substrate concentration (4MC) with Cu-Pep1 (A) and Cu-Pep2 (B) complex 1:1. $C_{Cu(II)}$ = 25 μM and $C_{peptide}$ = 25 μM, [4MC] = 0-4 mM, 50 mM HEPES pH 7.4, T = 293.2 K.

**Table S1. Kinetic Parameter for the Catalytic Oxidation of 4MC in aqueous solution at pH 7.4 in Hepes 50 mM at T = 298.2 K.**

| | $k_{cat}$ ($s^{-1}$) | $k_{cat}/K_M$ ($M^{-1}$ $s^{-1}$) | $K_M$ (mM) |
|---|---|---|---|
| Pep1 | 0.004(1) | 7.22(1.94) | 0.59(9) |
| Pep2 | 0.004(1) | 7.59(1.92) | 0.54(8) |

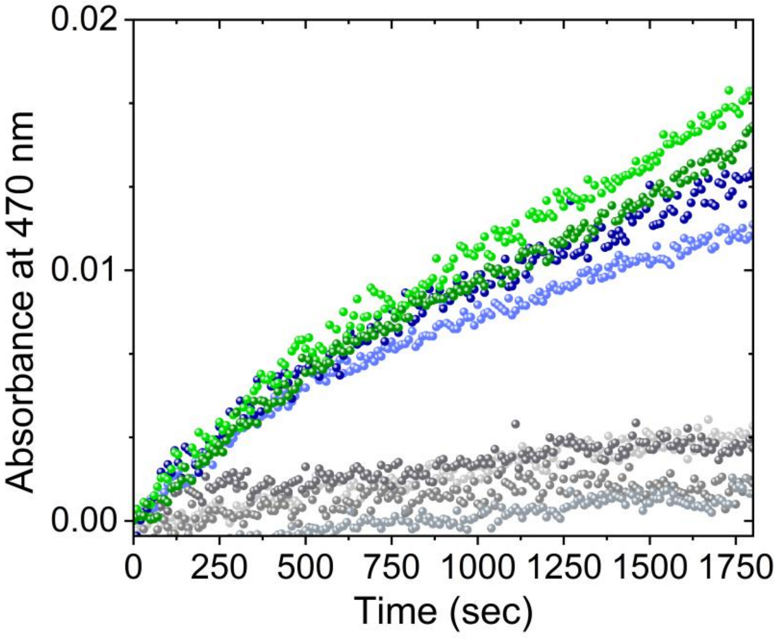


**Figure S9** UV-Vis kinetics of oxidation of L-/D- DOPA with Pep1 (blue) and Pep2 (green) and control in light grey L-/D-DOPA alone and in the darker grey L-/D- DOPA with copper. (50mM HEPES pH 7.4, PepX 25µM, Cu 25µM, L-/D- DOPA 3 mM).

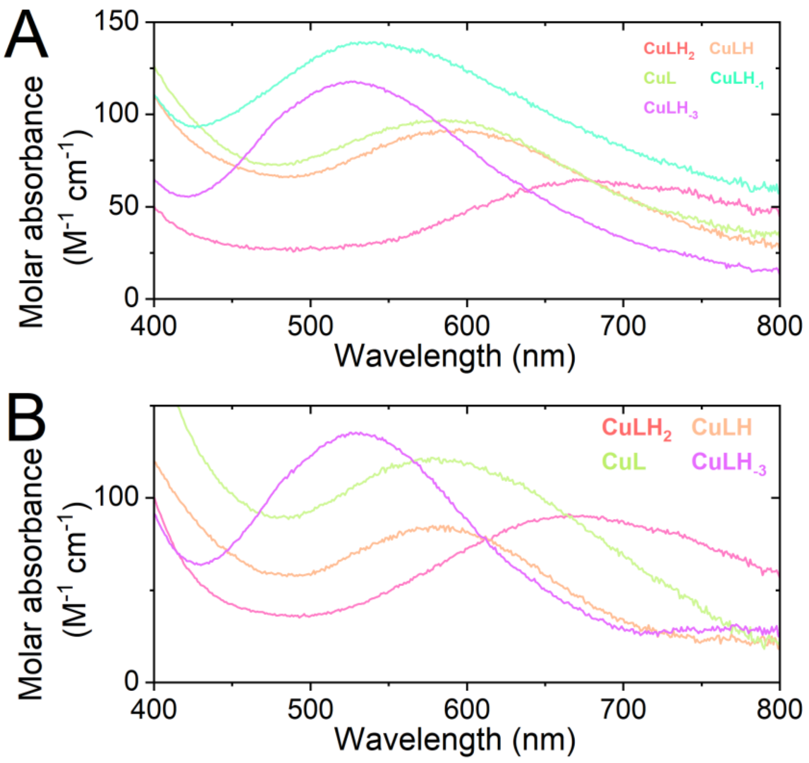


**Figure S10** Calculated UV-Vis spectra for the pH titration of Cu(II):PepX systems (Pep1 in panel A, and Pep2 in panel B). Cu/PepX = 1:1.25) based on potentiometric complexation model, at $T$ = 298.2 K and $I$ = 0.1 M (KCl). $C_{Cu}$ = 0.32 mM, [PepX] = 0.4 mM.

**Table S2. Experimental and calculated absorption of copper(II) complexes of PepX (X=1 or 2) in aqueous solution (T = 298.2 K, *I* = 0.1 M in KCl).**

| Species | Pep1 $[H_5L]^{3+}$ | | | Pep2 $[H_5L]^{3+}$ | | |
|---|---|---|---|---|---|---|
| | Experimental | Billo's | HypSpec2014 | Experimental | Billo's | HypSpec2014 |
| $[Cu(LH_2)]^{2+}$ | 650 | 650 | 679 | 663 | 650 | 675 |
| $[Cu(LH)]^{+}$ | 608 | 583 | 591 | 609 | 583 | 587 |
| [Cu(L)] | 584 | 588 | 586 | 583 | 588 | 581 |
| $[Cu(LH_{-1})]^{-}$ | 523 | 523 | 527 | 530 | 523 | 532 |
| $[Cu(LH_{-2})]^{2-}$ | 523 | 523 | --- | 530 | 523 | --- |
| $[Cu(LH_{-3})]^{2-}$ | 523 | 523 | 526 | 530 | 523 | 533 |


(1) Gans, P.; Sabatini, A.; Vacca, A. Investigation of Equilibria in Solution. Determination of Equilibrium Constants with the HYPERQUAD Suite of Programs. *Talanta* **1996**, *43* (10), 1739–1753. https://doi.org/https://doi.org/10.1016/0039-9140(96)01958-3.

(2) Gans, P.; Sabatini, A.; Vacca, A. Determination of Equilibrium Constants from Spectrophometric Data Obtained from Solutions of Known PH : The Program PHab. *Ann Chim* **1999**, *89*, 45–49.

(3) Sokołowska, M.; Bal, W. Cu(II) Complexation by "Non-Coordinating" N-2-Hydroxyethylpiperazine-N'-2-Ethanesulfonic Acid (HEPES Buffer). *J Inorg Biochem* **2005**, *99* (8), 1653–1660. https://doi.org/10.1016/j.jinorgbio.2005.05.007.

(4) Alies, B.; Badei, B.; Faller, P.; Hureau, C. Reevaluation of Copper(I) Affinity for Amyloid-β Peptides by Competition with Ferrozine—An Unusual Copper(I) Indicator. *Chemistry – A European Journal* **2012**, *18* (4), 1161–1167. https://doi.org/10.1002/chem.201102746.
